# A Framework for Monitoring Contaminants in Decentralized Agricultural Systems: Leveraging Stakeholder Incentives in Low-Resource Settings

**DOI:** 10.3390/toxins18080335

**Published:** 2026-08-01

**Authors:** Lee E. Voth-Gaeddert, Hannah Glesener, Gabriela Montenegro-Bethancourt

**Affiliations:** 1 Biodesign Center for Health Through Microbiomes, Arizona State University, Tempe, AZ 85281, USA; hglesene@asu.edu; 2 Center for Indigenous Health Research, Wuqu’ Kawoq|Maya Health Alliance, Tecpan, Chimaltenango 04006, Guatemala; gmont.bt@gmail.com; 3 School for Engineering of Matter, Transport, and Energy, Arizona State University, Tempe, AZ 85281, USA

**Keywords:** decentralized agricultural systems, supply chain monitoring, aflatoxin, low-resource settings, smallholder agriculture, Guatemala

## Abstract

Decentralized agricultural systems (DAS) are essential to food security for millions of people in low-resource settings, yet most existing contaminant monitoring frameworks have been designed for centralized supply chains. This paper proposes a structured framework for integrating contaminant monitoring into DAS by (1) mapping the diverse supply chain structures and stakeholders that constitute these systems; (2) analyzing stakeholder motivations, constraints, and value propositions for monitoring adoption; and (3) identifying efficient monitoring points where these value propositions align with product quality improvement. The framework is organized around four core principles: tiered monitoring matched to supply chain position and stakeholder capacity, monitoring at stakeholder handoff points rather than within individual segments, incentive structures that make monitoring self-sustaining, and capacity building and governance as durable infrastructure. The framework is illustrated through the case of maize and aflatoxin contamination in Guatemala. It remains a conceptual framework, however, and its principles are intended to be generalizable across crops, contaminants, and geographies rather than demonstrated to be so. Empirical validation across these dimensions remains a necessary next step. The central argument is that through this framework, small, strategically placed incentives at high-leverage points in decentralized supply chains can shift system-wide quality outcomes. This can occur without requiring top-down regulatory enforcement, which is often neither feasible nor effective in these contexts.

## 1. Introduction

Contamination of staple crops can pose persistent and disproportionate threats to public health in low-resource settings [1,2]. Regulatory bodies and food safety organizations primarily focus their surveillance on mycotoxins, heavy metals, pesticide residues, and pathogenic bacteria that frequently compromise the integrity of these essential food sources [3]. For example, aflatoxins, a type of fungal mycotoxin produced by *Aspergillus flavus* and *A. parasiticus*, are potent carcinogens associated with hepatocellular carcinoma, immune suppression, impaired child growth, and acute poisoning events [4,5,6,7]. These toxins contaminate dietary staples such as maize, groundnuts, and sorghum and are proliferated by poor post-harvest handling, storage practices, and environmental conditions [8,9,10]. For these fungal toxins, the greatest burden falls on food-insecure populations, who often have limited economic resources to discard contaminated grain and instead consume or blend damaged kernels with otherwise clean stock [11,12].

Effective monitoring is fundamental to any strategy aimed at reducing contaminant exposure; however, the existing frameworks for systematic monitoring were largely developed for centralized agricultural supply chains in high-income settings [13]. For example, Fumagalli and colleagues have proposed a framework for mycotoxin monitoring that accounts for infrastructure, laboratory capacity, and regulatory architecture, but within industrialized food systems [13]. Such frameworks presuppose formalized supply chains with high traceability, consolidated processing nodes, and enforceable regulatory standards, conditions that are less common in decentralized agricultural systems (DAS) that supply staple foods to much of the world’s most vulnerable populations [14,15].

These existing approaches differ from the framework proposed here in their underlying assumptions. Hazard Analysis and Critical Control Points (HACCP) systems, for example, presuppose defined process steps and enforceable critical limits, and mycotoxin-management frameworks developed for industrialized chains assume consolidated processing and traceability from farm to consumer. The literature on informal markets and food-safety governance suggests that these assumptions hold weakly in DAS, where oversight is fragmented and traceability is limited. The framework proposed here differs by placing monitoring at stakeholder handoff points and by aligning incentives with the actors who must perform it, rather than by relying solely on formalized enforcement.

DAS, as defined in this paper, are localized networks in which food is cultivated, processed, and consumed within a defined geographic area, often at the community or household level, without relying exclusively on large-scale production or centralized distribution infrastructure [16,17]. These systems may include subsistence farming, a common term in agricultural development programs, but DAS are broader. DAS encompass a range of configurations including semi-commercial smallholders who sell surplus crops at local markets, informal cooperatives that aggregate grain for collective sale, intermediary-mediated supply chains in which middlemen purchase from multiple producers and redistribute to regional markets, and institutional supply chains through which government programs procure from smallholders for school feeding or social assistance [18]. What unifies these diverse systems is that the operational burden of quality management falls primarily on individual households and small-scale stakeholders who often lack the resources, technical capacity, and market incentives needed to implement systematic monitoring.

To avoid conceptual overextension, DAS are further defined here by inclusion and exclusion criteria. The inclusion criteria are localized production, processing, and consumption within a defined geographic area; an operational burden of quality management that rests on households and small-scale actors; coordination through informal social networks; and limited end-to-end traceability. Fully vertically integrated or contract-farming chains with centralized processing and enforceable standards fall outside this definition. These criteria describe a system’s predominant character rather than a strict category, since real systems occupy a continuum between centralized and decentralized forms.

The purpose of this paper is to present a framework for integrating or strengthening contaminant monitoring into DAS in low-resource settings. The framework proceeds in three stages: (1) a systematic mapping of the diverse supply chain structures and stakeholders that make up these systems; (2) an analysis of motivations, constraints, and value propositions that shape monitoring adoption; and (3) the identification of efficient monitoring insertion points and corresponding approaches where these value propositions align with crop-based quality improvements. The framework is illustrated using maize systems in Guatemala, where aflatoxin contamination intersects with high rates of chronic malnutrition, traditional post-harvest practices, and complex informal supply chains. However, the principles articulated here are intended to be generalizable beyond this specific crop, contaminant, and geography. This paper is a conceptual framework paper. It proposes and structures a monitoring approach and illustrates it with a case, and it does not report primary empirical data or a validated analytical protocol; the field screening tools described are intended to triage product lots toward confirmatory laboratory testing rather than to replace it.

## 2. Mapping DAS Supply Chain Structures

Using maize in Guatemala as an illustrative context, we (1) characterize the structural features of DAS, (2) identify 10 common supply-chain scenarios and group them into five functional categories, and (3) identify the critical control points at which contamination risk is introduced, amplified, or potentially mitigated.

### 2.1. Structural Contrasts Between Centralized and Decentralized Agricultural Systems

**Figure 1:**
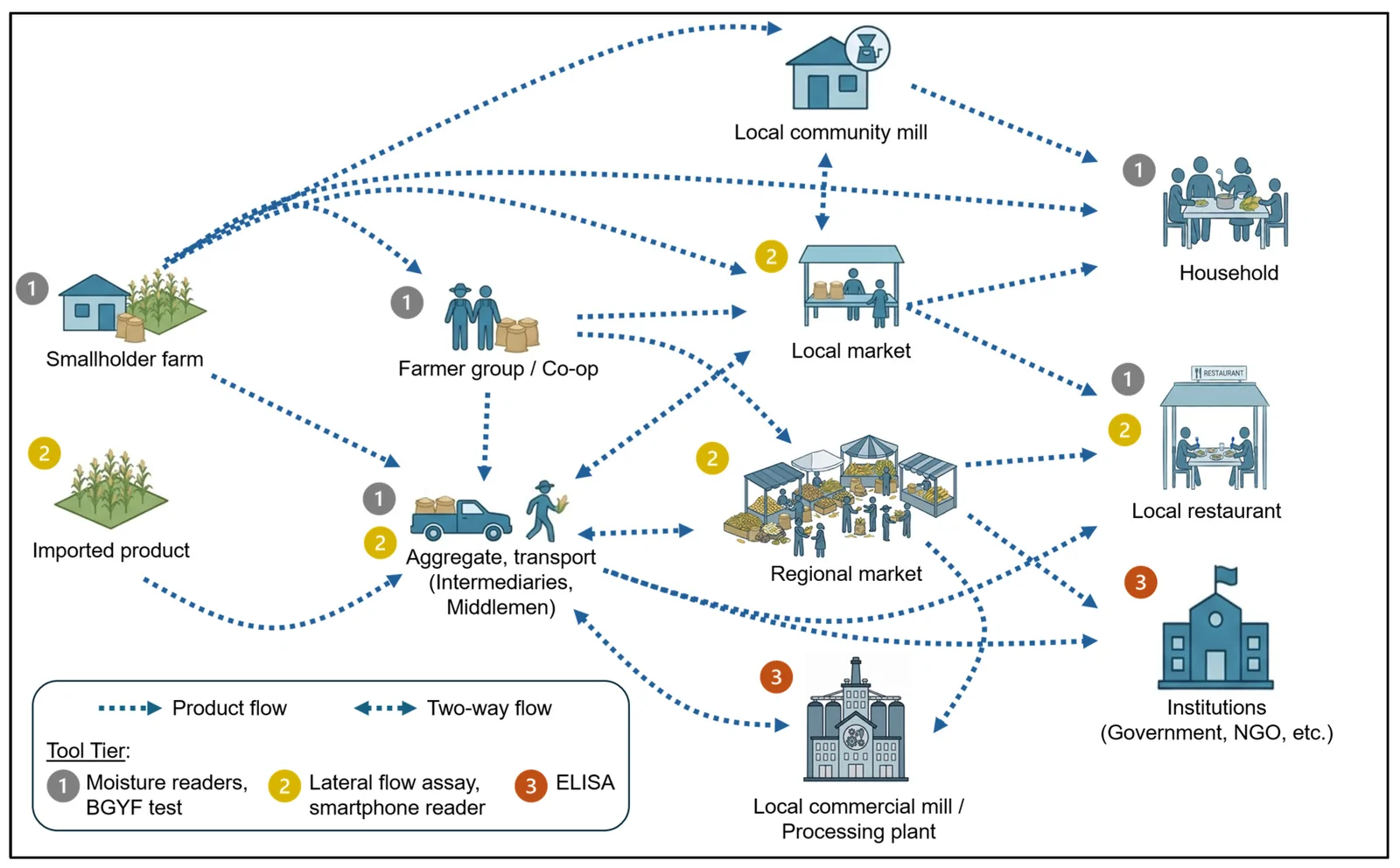
Depiction of the components of a DAS with key stakeholders, product flows (dotted lines), and monitoring points (numbers). Storage durations, volumes, and product flows shown are illustrative expert estimates for the Guatemalan context rather than measured values.

Centralized agricultural systems (CAS) and DAS differ across four interrelated dimensions, namely physical infrastructure, economic structure, social dynamics, and governance, that collectively shape how crop quality is managed and where monitoring can be effectively positioned (see Supplementary Martials and Figure 1). Physically, CAS rely on large-scale, purpose-built infrastructure: industrial processing facilities, controlled-environment storage, and formalized transport networks managed by specialized personnel operating under standardized procedures [19,20]. DAS, by contrast, are made up of dispersed, household-owned components: small agricultural plots, traditional drying areas such as patios or kitchen lofts (*tapancos*), diverse storage containers, and informal transport by foot, motorbike, or small truck. Furthermore, the operational burden of post-harvest processing, handling and storage falls primarily on individual farmers and households [11,21]. Economically, CAS are characterized by centralized finance, formal contracts, and access to credit and insurance, with brand reputation providing a direct market incentive for consistent quality [20,22]. In contrast, DAS rely primarily on household-level investment in operation and maintenance, with food security and surplus sales serving as the main economic drivers. Intermediaries who aggregate grain from multiple small producers are typically motivated by price differentials and rapid turnover rather than by crop quality differentiation [23,24].

The social and governance dimensions further highlight key differences. CAS operates through formal institutional relationships between corporate entities and supply chain partners, whereas DAS rely primarily on informal social networks including kinship ties, neighborhood relationships, and loosely organized farmer groups, for knowledge exchange, resource sharing, and collective action [25,26]. Although these networks provide important resilience and adaptive capacity, they often lack the formal quality control mechanisms or standardized testing capabilities required for systematic crop quality assurance. From a governance perspective, CAS function with defined regulatory frameworks, supported by established standards, certification systems, and enforcement mechanisms that enable high levels of traceability from farm to consumer. DAS often function within weak or fragmented regulatory or enforcement ecosystems, where oversight is limited, standards are inconsistently defined or poorly enforced, and agricultural policies frequently prioritize centralized systems [27]. As a result, traceability in DAS is substantially constrained, particularly when intermediaries mix product from multiple sources, while incentives for quality improvement remain weak, with little or no market premium for higher-quality crops and quality assessments relying largely on trust and visual inspection. These characteristics should not be viewed as deficiencies to be corrected through the imposition of centralized models; rather, they represent the structural conditions within which any effective monitoring framework for DAS must be designed to operate.

In practice, centralized and decentralized systems are endpoints of a continuum rather than a strict dichotomy. Many real chains are hybrid; for example, smallholder produce may enter formal processing, and informally aggregated grain may be sold into institutional procurement. Traceability in these hybrid chains is often partial rather than absent. The four dimensions above are therefore best understood as axes along which a given system can be positioned, and the framework is designed to accommodate this variation.

### 2.2. Typology of DAS Supply Chains

Field observations and prior literature suggest that DAS supply chains are not uniform but instead encompass a range of configurations that differ in length, complexity, scale, and degree of formality. We identify ten common scenarios, grouped according to their structural similarities. Table 1 presents the ten supply chain scenarios with their associated steps, key stakeholders, typical scale and timing, and prevailing crop quality control practices.

The typology was developed from field observation in Guatemalan maize systems, prior published literature on those systems, and iterative expert synthesis. It represents an expert typology rather than a systematic survey, and it has not yet undergone formal stakeholder validation. Participatory validation of the typology with local actors is a stated next step.

**Household-terminal chains.** In Scenario 1 (direct household consumption), smallholder farmers cultivate, harvest, dry, store, and consume maize entirely within the household, with no external transactions. Quality management rests solely with the household and storage may extend from weeks to months, depending on consumption needs. As a result, quality outcomes depend entirely on growing quality, local drying practices, and storage conditions. This scenario represents the shortest supply chain, the highest individual operational burden, and the lowest point of external leverage for monitoring interventions.

**Farmer-to-market chains.** In Scenarios 2 (direct sales to local markets) and 3 (informal group aggregation for local markets), farmers, or small groups of neighboring farmers, transport maize to local or community markets for sale. These supply chains are short, typically spanning one to five days from source to sale, and operate on a relatively small scale, usually involving tens to hundreds of kilograms. Visual inspection often serves as the primary method of quality assessment. While group aggregation can improve transport efficiency and reduce transaction costs, it also introduces the challenge of mixing product from multiple producers, which can obscure the source of quality variation and reduce traceability. These supply chains are largely underleveraged for information dissemination [28]: Establishing simple norms of transparency, such as displaying moisture content or test results at the point of sale, could therefore represent a low-cost intervention with potentially high impact.

**Intermediary-mediated chains.** Scenarios 4 (“middleman” aggregation for local markets) and 5 (middleman aggregation for larger regional markets) introduce the intermediary as a central actor. Middlemen purchase maize directly from farms in bulk, often combining product from multiple producers, and in some cases, mixing it with imports from neighboring countries, before reselling it to local or regional markets. These supply chains typically involve medium-to-large volumes (upwards of several tons), and storage periods that may extend to two weeks or more in intermediary warehouses. As a result, traceability is substantially reduced. The intermediary node represents both the point of greatest quality risk, due to source mixing and extended storage, and the point of greatest potential leverage, because a large volume of product passes through a single actor.

**Processing-destination chains.** Scenarios 6 (sales to local restaurants and *tortillerías*), 7 (supply to local milling or processing plants), and 8 (supply to larger food processing facilities) involve the delivery of product, such as maize, to entities that perform on-site processing. *Tortillerías* and small restaurants purchase product in frequent, small batches and process maize through nixtamalization, which can reduce aflatoxin levels by an estimated 60–70% under favorable conditions. However, this is not a complete remediation strategy, and its effectiveness depends on specific processing parameters. Local mills may apply basic quality checks, such as moisture content and visible mold, prior to milling, whereas larger processing facilities are more likely to implement formal quality control procedures, although the consistency of enforcement varies. These supply chains introduce both opportunities for partial remediation and additional points of risk, with the degree of quality control generally increasing according to facility size and market exposure.

**Institutional and cross-border chains.** Scenario 9 (institutional or government procurement and distribution) involves farmers or aggregators selling their product to public institutions for use in social programs, such as school feeding initiatives. These supply chains may require prolonged storage in government warehouses, raising mold development risk (in the case of maize, and consequently aflatoxin contamination) if storage conditions are poorly managed. Although procurement processes may formally require quality testing, enforcement is often inconsistent. Scenario 10 (cross-border trade) introduces the product from neighboring countries, particularly Mexico in the context of Guatemalan supply chains, which may enter through informal or weakly regulated channels. When imported crops are mixed with domestic production, local regulatory oversight is further reduced and traceability is difficult to maintain.

### 2.3. Critical Control Points for Contamination

The logistical challenge of monitoring is complicated by the distinct behavior of key contaminants of concern. Unlike chemical contaminants that are effectively “dosed” in the field and remain relatively static (e.g., heavy metals) or gradually decline in post-harvest (e.g., pesticide residues), fungal toxins and associated pathogens can continue to develop and proliferate throughout the supply chain. For example, initial fungal contamination introduced at one stage may intensify during subsequent stages, while mixing at aggregation nodes can diffuse responsibility and obscure the original source of contamination. Identifying the critical control points at which contamination is introduced, amplified, or can be mitigated is therefore essential for designing efficient monitoring systems. Below, we examine these critical control points in the context of fungal toxins.

These control points can be prioritized using the logic of HACCP, adapted to the constraints of DAS. Three criteria determine priority here: the throughput volume passing through a node, since aggregation concentrates both contamination risk and monitoring leverage at a single actor; storage duration and water activity, which drive accumulation; and the irreversibility of source-mixing, after which lot-level information is lost. On these grounds, the aggregation handoff is the highest-priority control point. A full quantitative risk ranking would require the field data identified in Section 6 as a research priority.

**Pre-harvest.** Field conditions, crop variety, planting density, and exposure to pest and drought stress influence susceptibility to fungal colonization. Biocontrol products such as Aflasafe, competitive exclusion agents designed to suppress aflatoxin-producing fungal strains, have demonstrated substantial reduction in pre-harvest contamination in field trials [29]. However, adoption in DAS remains limited due to barriers related to cost, distribution, and producer awareness.

**Harvest and drying.** Moisture mishandling during harvest and drying is one of the most critical risk factors for post-harvest aflatoxin accumulation [8,30]. Delayed harvest, rain exposure during drying, and insufficient drying before storage create conditions highly favorable to *Aspergillus* growth. Traditional sun-drying practices are highly variable in effectiveness and dependent on weather conditions. Improved drying techniques, including elevated drying racks, solar dryers, and the use of tarpaulins, can substantially reduce moisture-related risk.

**Storage.** On-farm storage is a primary control point for aflatoxin management. Maize stored at moisture contents above approximately 13%, particularly under warm, humid, and poorly ventilated conditions, is highly susceptible to mold growth and progressive mycotoxin accumulation [31,32]. Storage duration compounds risk: grain retained for several months, as is common for household consumption, may accumulate substantially higher contamination levels than grain sold shortly after harvest. Hermetic storage technologies, including sealed bags and metal silos, reduce both moisture-related deterioration and pest-associated contamination, improving storage safety and grain quality [32,33].

**Transport and aggregation.** Transport conditions (humidity, rain exposure, container contamination from storage containers or vehicles) contribute to aflatoxin accumulation. Another key concern at this stage is the aggregation of grain from multiple sources by intermediaries. Mixing obscures the quality of individual lots, concentrates contamination risk across the combined volume, and eliminates the traceability necessary for accountability. The aggregation node is therefore a critical point not only for contamination risk, but also for monitoring design: it is often the last opportunity to assess grain quality before individual lots lose their identity.

**Processing.** Processing steps such as nixtamalization, milling, and sorting can partially reduce contaminant levels, but their effectiveness is variable and they do not eliminate risk [34,35]. Processing should be understood as a supplementary mitigation measure rather than a substitute for upstream quality management.

The key structural insight for monitoring design is that the most efficient monitoring points are located at transitions between stakeholders—the handoff points where product changes ownership. The rationale and placement logic for this insight are developed as Principle 2 (Section 4.2).

## 3. Stakeholders: Motivations, Constraints, and Leverage Points

The adoption and long-term sustainability of any monitoring system in DAS depends on its alignment with the motivations of the stakeholders responsible for implementing it. This section examines the major stakeholder groups in DAS, using maize as an illustrative case, organized not by their position in the supply chain, but by their relationship to the value proposition that monitoring must provide (see Table 2).

### 3.1. Producers

Smallholder farmers cultivate, harvest, dry, and store maize, and may sell surplus production to markets or intermediaries. Their primary motivations are household food security and income generation from surplus sales, and their interest in minimizing post-harvest losses by mold and pests is genuine but constrained by limited access to improved storage technologies, financial resources, knowledge of effective mitigation practices, and available time. For this group, the value proposition for adopting monitoring systems must center on tangible economic benefits: price premiums for quality-verified grain, reduced post-harvest losses through earlier detection of contamination, and improved access to higher-value market channels. Household members, including smallholder farmers, who store and prepare maize for family consumption are primarily motivated by family welfare, food security, and cost savings, but often have limited time, incentive, or technical capacity for routine self-testing. They are vulnerable to weather variability, pest damage, and moisture-related deterioration in stored grain, and may lack access to information on safe moisture thresholds. For this group, monitoring value lies in simple, low-cost tools (e.g., moisture indicators) and practical, actionable guidance that can be integrated into existing household routines without imposing significant additional labor or financial burden.

### 3.2. Aggregators and Intermediaries

Intermediaries (“middlemen”) travel to farms to purchase maize in bulk, transport it, and frequently mix grains from multiple sources for resale. Their primary motivations are profit from price differentials and logistical efficiency, and their time horizon is typically short: with rapid turnover prioritized over quality. In most cases, they receive little reward for handling higher-quality grain, while accountability for downstream contamination remains minimal. Informal farmer groups and cooperatives similarly aggregate maize from multiple producers for collective sale, improving transport efficiency and negotiation power but often lacking formal quality control systems. Transporters are the individuals responsible for moving maize between supply chain nodes and are generally compensated per trip (often by an intermediary or co-op) or by volume, with no direct incentive to protect product quality during transit. For these stakeholders, the value proposition for monitoring must be framed around competitive advantage and risk reduction: for example, “Verified Seller” certification that provides preferred supplier status, reduced rejection rates at processing facilities or institutional buyers, and access to purchasers willing to pay premiums for traceable, quality-assured lots. Even small price differentials can meaningfully shift purchasing behavior when margins are narrow.

### 3.3. Processors and Retailers

Tortillerías and local restaurants purchase maize in frequent, small batches and process it on-site. Their primary motivations are maintaining a consistent supply, preserving customer trust and ensuring product quality, but they operate under thin profit margins and face limited consumer demand for formal testing or certified inputs. Local and community mills provide milling services and may also purchase maize for flour or masa production. Although they often lack testing equipment and training, they handle sufficient volume to function as practical quality checkpoints if appropriately supported. Larger commercial processors are more likely to implement formal quality control procedures, but enforcement varies and cost pressure can limit sustained investment. For processors and retailers, the value proposition of monitoring lies in product differentiation and reputational advantage, even within informal markets. A tortillería known for using tested maize, or a local mill able to certify its flour or masa, can create a meaningful competitive distinction that translates into customer loyalty and greater willingness to pay.

### 3.4. Institutional Buyers and Enabling Actors

Government agencies procure maize for social programs and may also manage storage and distribution through public warehouses. Their primary motivations are public welfare, food safety, and program credibility, but they are often constrained by bureaucratic processes, limited technical capacity, and inconsistent enforcement resources. NGOs and development organizations frequently support these systems by providing training, strengthening cooperatives, and channeling donor funding toward improved practices and quality management. However, they face the persistent challenge of sustaining interventions beyond the duration of individual projects. For these institutional buyers, monitoring provides value through verifiable impact metrics and program credibility; demonstrable evidence that procurement standards are met and that beneficiaries, such as schoolchildren or food aid recipients, are receiving safe food. For enabling actors (e.g., NGOs), the value lies in scalable implementation models and measurable outcomes that justify continued investment and long-term support.

### 3.5. The Incentive Gap

The analysis above reveals a systemic incentive gap: the stakeholders who most directly influence quality outcomes, such as producers and intermediaries, often capture insufficient value from quality improvement to justify the cost of monitoring, while those who benefit most from safer, higher-quality grain (consumers, institutional buyers) remain economically distant from the points of quality control and are unable to transmit quality signals through the market [36,37]. This misalignment represents a fundamental market failure and is the central challenge that any monitoring framework must address [38,39]. The objective, therefore, is not to impose monitoring as an unfunded compliance requirement, but to redesign incentive structures so that monitoring becomes an economically rational and self-sustaining practice for the actors responsible for implementing it. The stakeholder motivations described in this section are analytic rather than empirically measured here, and the strongest behavioral claims (i.e., willingness to test, response to price premiums, and reputational benefit) are best treated as hypotheses for empirical validation.

## 4. A Framework for Monitoring Integration in DAS

**Figure 2:**
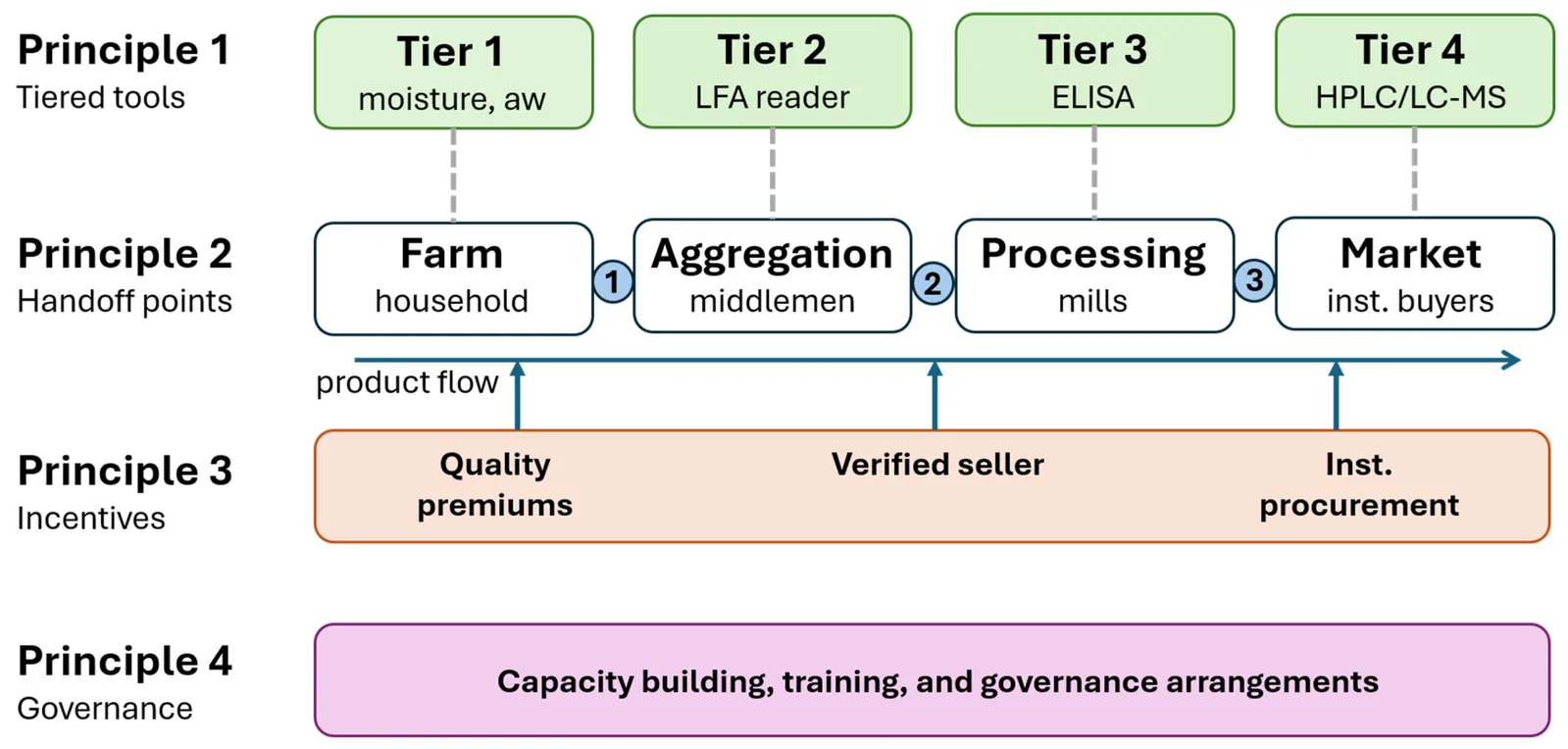
Summary schematic of the four-principle monitoring framework. Tiered monitoring tools (Principle 1) are matched to positions along the supply chain; numbered points mark monitoring at stakeholder handoff points (Principle 2); incentive structures (Principle 3) make monitoring self-sustaining; and capacity building and governance (Principle 4) provide the durable foundation.

To effectively integrate contaminant monitoring into decentralized agricultural supply chains, we propose a four-principle framework. This framework synthesizes the structural characterization of DAS, the supply chain mapping, and the stakeholder analysis into a set of operational principles designed to make monitoring feasible, adoptable, and sustainable in low-resource settings. Figure 2 summarizes the four principles together with the tiered-monitoring and handoff-point logic that links them.

### 4.1. Principle 1: Tiered Monitoring Matched to Chain Position and Stakeholder Capacity

No single monitoring technology is appropriate across all nodes of a decentralized supply chain. A tiered approach ensures the precision, cost, and technical requirements of monitoring tools are aligned with the capacity, incentives, and decision-making needs of stakeholders at each point in the supply chain. This tiering does not imply that established, gold-standard methods are technically inadequate. Rather, these methods are often inaccessible at most nodes because of cost and instrumental availability, and a tiered design reserves them for the nodes and lots where they are justified while enabling lower-cost triage upstream.

**Tier 1—Field and household screening.** At the farm or household level, monitoring tools must be inexpensive, rapid, and usable without specialized training. Moisture readers provide a practical first-line screen by allowing farmers to assess whether grain has been dried to safe storage thresholds, generally below 13% moisture content. Water activity, rather than moisture content alone, governs the risk of storage-fungal growth, and low-cost water-activity indicators may complement moisture readers where available. Bright greenish-yellow fluorescence (BGYF) testing using a portable blacklight offers an additional low-cost visual screen for potential *Aspergillus* infection. These tools do not directly quantify contaminant levels but support a critical binary decision: whether the grain is sufficiently safe for storage, or whether it requires further drying, sorting, or diversion before use. Visual and BGYF screening are error-prone and cannot classify toxin levels; they support this binary handling decision (i.e., dry further or divert versus store) rather than a determination of contamination, and screen-positive or uncertain lots are routed to higher tiers.

**Tier 2—Aggregation and market-level rapid testing.** At aggregation points, cooperatives, and local markets, semi-quantitative monitoring tools are more appropriate. Lateral flow assays (LFAs) and smartphone-based readers can provide rapid presence/absence detection or approximate concentration estimates for key contaminants. In this context, the smartphone-based reader quantifies the test line of the LFA, and it does not refer to image-based scanning of grain for discoloration. Toxin risk should not be inferred from visible damage, because non-toxigenic and toxigenic fungi may produce macroscopically identical symptoms. These tools are suitable for use by intermediaries, cooperative representatives, market vendors, and agricultural extension agents conducting testing at local or weekly markets. The primary decision supported at this tier is whether to accept, reject or reprice a lot, thereby enabling quality-based price differentiation.

**Tier 3—Processing and institutional-level semi-quantitative testing.** At larger mills, commercial processing facilities, and government procurement warehouses, validated enzyme-linked immunosorbent assays (ELISA) provide accessible, semi-quantitative testing that can flag suspect lots and prioritize them for definitive analysis. Unlike chromatographic methods, ELISA does not require highly specialized laboratory infrastructure and can be implemented in modestly equipped regional laboratories or through mobile testing units, making it a practical option for decentralized monitoring systems. ELISA is susceptible to matrix interference and cross-reactivity, and it is therefore best treated as a semi-quantitative screening step rather than a confirmatory method. Calibration, quality control, and a maintained, reagent cold chain are required, and screen-positive results are routed to Tier 4 for confirmation.

**Tier 4—Reference and surveillance.** High-performance liquid chromatography (HPLC) and liquid chromatography-tandem mass spectrometry (LC-MS/MS), conducted in regional reference laboratories, serve the functions of monitoring for public health surveillance, calibration and validation of lower-tier methods, and definitive quantification for research and regulatory purposes. A “traveling” ELISA laboratory, a mobile unit that provides scheduled, localized semi-quantitative testing on a rotating basis, can help bridge the gap between Tier 3 and Tier 4 in settings where permanent laboratory infrastructure is not feasible. The design parameters for such a unit include scheduled routing among market and aggregation sites, service frequency tied to harvest and market calendars, intermediate depots for reagent and sample logistics, staffing and training, reagent cold chain and stability, calibration against reference methods, and an indicative cost per test; these parameters can be optimized using methods developed for mobile service units with intermediate depots [40]. Quantitative PCR (qPCR) may complement this tier for research and surveillance by estimating mycotoxigenic fungal biomass, although it quantifies fungal presence rather than toxin concentration and is therefore not proposed as a routine monitoring tier.

The tiered structure ensures that each node in the supply chain has access to a monitoring tool that is appropriate to its resources, technical capacity, and decision-making needs. It also creates a quality-information gradient in which testing precision increases as the product moves downstream and approaches final consumption, reflecting both the rising economic value of the product and the increasing public health consequences of contamination at later stages.

### 4.2. Principle 2: Monitoring at Stakeholder Handoff Points

Monitoring is most efficient and least burdensome when it occurs at the moments when product changes hands between stakeholders. At these handoff points, the buyer has a natural economic incentive to verify quality, as they are committing resources to the purchase, while the seller has an incentive to demonstrate quality in order to secure the sale and maximize price. The transaction itself creates a set timing in which testing is expected, justified, and directly actionable.

This principle directs monitoring resources toward transitions rather than interiors: the point at which an intermediary purchases from a farmer (Tier 1–2 testing), the point at which a co-op aggregates member contributions (Tier 2), the point at which a mill receives grain from an intermediary or aggregator (Tier 2–3), and the point at which a government warehouse receives a procurement lot (Tier 3). Positioning monitoring at these handoff points distributes the responsibility for quality assurance across the supply chain rather than concentrating it on the least-resourced actor, the farmer. It also leverages the economic dynamics of the transaction (the buyer’s need to avoid contaminated products and the seller’s incentive to secure a fair price) to make monitoring self-reinforcing. This principle also addresses the aggregation problem directly by placing monitoring at the aggregation handoff, before mixing occurs and lot-level quality information is lost.

Two practical constraints shape monitoring at these points. First, mycotoxins are distributed heterogeneously within a lot, so representative multi-increment (composite) sampling is required, particularly for mixed lots at aggregation nodes. Second, field screening tools have finite detection limits and are subject to false-negative and false-positive results and to operator variability. For these reasons, screening at a handoff is treated as a triage step, and screen-positive or borderline lots are routed to confirmatory testing at the next tier rather than accepted or rejected on the screen alone.

### 4.3. Principle 3: Incentive Structures That Make Monitoring Self-Sustaining

Monitoring will be adopted at scale only if it is economically rational for the actors who must perform it. Externally funded pilot programs can demonstrate feasibility, but long-term sustainability depends on embedding monitoring within incentive structures that create self-reinforcing returns.

**Market-based incentives.** Price premiums for quality-verified grain create a direct financial return on monitoring investments, and these premiums need not be large: even modest differentials (5–10%) can influence crop sorting and handling behavior when profit margins are thin [29,41]. “Verified Seller” certification programs and “Quality Maize Bonus” schemes formalize these incentives and create a reputational asset for participants [41,42]. In this model, farmers, intermediaries, cooperatives, and mills can be recognized as “verified” stakeholders based on regular testing, traceability, and adherence to quality protocols. The status could be granted by trusted institutions such as co-ops, large buyers, certification bodies, or public agencies, and maintained through periodic verification. The USDA’s aflatoxin certification programs for export commodities demonstrate that certification mechanisms can successfully incentivize quality at the producer level; the key challenge is adapting similar mechanisms to decentralized and informal markets [43].

As an illustrative example, a per-lot premium becomes economically rational when the expected premium exceeds the sum of the per-lot testing cost, the expected cost of rejected or repriced lots, and transaction and delay costs. The break-even premium therefore rises with test cost and with the rejection rate, and falls as lot value increases. This relationship is presented as an order-of-magnitude model to structure future empirical work rather than as an empirical result.

**Informational incentives.** Transparency in product quality is itself a form of incentive. Making quality visible at point of sale, through posted moisture content, rapid test results, or simple visual indicators such as green/yellow/red signage, can reduce information asymmetry between sellers and buyers. Mobile platforms that share test results strengthen trust and accountability across the supply chain. In informal markets where quality is often judged only by appearance or personal trust, even modest increases in transparency can be market-transforming. However, information visibility alone may not overcome entrenched practice, because habitual decision-making and status quo bias can constrain responses to new information [44]. Informational incentives are therefore likely to be most effective when paired with a concrete transactional or financial trigger, as in the handoff-point and procurement mechanisms.

**Community-driven incentives.** Rotating testing services at weekly markets, supported by agricultural extension agents, can provide voluntary aflatoxin testing alongside small financial incentives or vouchers for producers whose grain meets quality thresholds. Success stories and peer recognition reinforce adoption, while awareness campaigns highlight both economic benefits (e.g., reduced post-harvest losses, improved market access) and health benefits that help shift community norms toward quality as a shared value.

**Institutional incentives.** Government procurement programs, such as Guatemala’s School Feeding Law, which requires that 50% of food purchases come from local smallholder farmers, can serve as a powerful lever for raising product quality [43,45]. By incorporating aflatoxin testing requirements in procurement standards, governments create a quality-demanding market channel that rewards producers who comply. NGO procurement initiatives that incorporate quality metrics into their own buying criteria can generate similar demand-side incentives.

**Financial incentives.** Microfinance products, microinsurance, or targeted subsidies around post-harvest investments (hermetic storage bags, improved silos, drying equipment) can help reduce the upfront cost barrier to improved handling. Crop insurance products that are linked to quality practices, along with group lending models operating through cooperatives, can further broaden access to quality improvement options for resource-constrained producers [46,47].

### 4.4. Principle 4: Capacity Building and Governance as Durable Infrastructure

Monitoring systems that rely on occasional training or on external project-based funding often fail once donor attention shifts. Similarly, the regulatory gap common in most DAS contexts is unlikely to be addressed in the near or medium terms through traditional top-down enforcement. Sustainable monitoring requires building capacity among supply-chain actors who are already embedded in the system and pairing this with governance arrangements that enable quality improvement from within communities.

**Extension agents as the monitoring backbone.** Agricultural extension services provide the most natural institutional platform for ongoing monitoring support. Extension agents trained in Tier 1–2 tools who maintain a regular presence at markets, co-ops, and aggregation points can deliver continuous technical assistance, conduct or supervise testing, and act as trusted mediators between producers and quality standards. Extension systems in many low-resource settings are under-resourced, however, and cannot serve as an analytical backbone on their own. Where extension capacity is too weak, embedded local service providers with independent incentives to keep operating may carry this function. In all cases, these actors conduct or supervise triage screening and route lots to confirmatory testing rather than performing definitive analysis in the field.

**Professionalization of local service providers.** Local millers, storage facility operators, and co-op managers process sufficient volume to operate as de facto quality checkpoints if they are equipped with basic testing tools and training. Investing in these actors, who have economic incentives to continue operating regardless of external funding, builds monitoring capacity that can persist beyond project timelines. Certification programs offer an additional layer of incentive.

**Participatory governance models.** Participatory Guarantee Systems (PGS) and Internal Control Systems (ICS) provide pathways to credible quality verification without the high cost and bureaucratic burden of formal third-party certification [48]. PGS involve local stakeholders directly in the verification process, strengthening community ownership of standards, while ICS establish structured internal quality assurance mechanisms within cooperatives or farmer groups.

**Progressive and enabling regulation.** Where regulatory frameworks exist, or are being developed, standards should be designed to be achievable in DAS contexts: graduated compliance thresholds, recognizing community-based certification mechanisms, and supporting implementation through subsidized testing and technical assistance rather than relying primarily on punitive enforcement. Regulations modeled on centralized systems and imposed without adaptation are likely to be ignored or to push DAS activities further into informality.

## 5. Illustrative Application: Maize and Aflatoxins in Guatemala

Guatemala illustrates the practical relevance of this framework. Nearly half of children under five are chronically malnourished, maize is the dietary staple, and production is overwhelmingly decentralized through smallholder systems rooted in Mayan agricultural heritage [11,49]. Aflatoxin contamination is well documented, driven by traditional post-harvest practices: sun-drying on patios, storage in ‘tapancos’, and the mixing of fungus-damaged ‘mulco’ with sound kernels as a matter of economic necessity [23]. All ten supply chain scenarios are present, with middlemen serving as pervasive intermediaries and cross-border imports from Mexico further complicating traceability [50,51]. Within this context, we highlight examples of how the four core principles could be applied.

First, the intermediary/middlemen purchasing node—where middlemen buy maize in bulk from farmers, often mixing sources immediately—represents the most consequential application of handoff-point monitoring (Principle 2). Deploying Tier 2 lateral flow assays at this transaction (Principle 1) would generate the quality information needed for a structural shift. Middlemen who can demonstrate that their aggregated stock was screened at the point of purchase gain access to a “Verified Seller” designation and preferred supplier status with mills, government programs, or institutional buyers. In this approach, the testing burden falls on the transaction rather than on the farmer, and the middleman’s investment is recouped through market differentiation, converting the chain’s highest-risk node into its primary quality gate.

Guatemala’s School Feeding Law, which mandates that 50% of food purchases come from local smallholders, creates an underutilized lever for Principle 3. Incorporating aflatoxin screening into procurement standards for school feeding programs would establish a high-quality-demanding market channel that rewards compliant producers and cooperatives with reliable institutional contracts, making quality economically rational rather than aspirational. On the governance and capacity side (Principle 4), Participatory Guarantee Systems applied to farmer cooperatives offer a culturally resonant alternative to third-party certification: community members conduct peer verification of post-harvest practices, building collective ownership of quality standards through social structures (e.g., trust networks, shared labor, and group reputation) that already function in highland Mayan communities. This approach embeds monitoring governance in institutions that predate and will outlast any externally funded project.

To make this approach sustainable, a multi-sectoral implementation framework is required that distributes functions across academia, government, private sector, and international cooperation while reinforcing shared accountability. Academia can lead the development and validation of low-cost aflatoxin testing protocols and participatory guarantee methodologies, while also generating local evidence on effectiveness and compliance incentives. Government institutions, particularly education and agriculture ministries, can institutionalize these standards within school feeding procurement systems, ensuring stable demand and embedding quality requirements into public purchasing rules. The private sector, including aggregators, millers, and input suppliers, chambers (agriculture, industry), and exporter associations, can be engaged through co-regulatory agreements that align commercial incentives with compliance, including preferential contracting for certified cooperatives and investment in post-harvest infrastructure. International cooperation can support initial capacity building, financing for testing infrastructure, and facilitation of knowledge exchange across regions. Together, these actors can also co-design awareness campaigns targeting farmers, school communities, and local authorities to build trust in certification systems and reinforce the value of food safety as both a public health and market advantage, thereby ensuring that quality assurance becomes a sustained institutional practice rather than a project-dependent intervention. The structural features that make these applications relevant to Guatemala (i.e., intermediary-mediated chains, weak enforcement, institutional procurement, and strong community social capital) are common across low-resource contexts globally, and the four principles are designed to be adapted to local configurations rather than applied as a template.

As a proposed design rather than an implemented study, a pilot could be structured as follows. Candidate districts would be selected for high maize dependence, documented aflatoxin burden, and the presence of active cooperatives and school-feeding procurement. Responsible actors would be assigned by tier: farmers and household members for Tier 1 screening, cooperatives and extension agents for Tier 2 at aggregation handoffs, and regional laboratories or the mobile unit for Tier 3 confirmation. Decision thresholds would follow regulatory limits for aflatoxin, with screen-positive lots routed to confirmation. The design would specify sampling frequency, a cost-sharing model across academia, government, the private sector, and international cooperation, adoption metrics, governance safeguards against masking, and measurable market and health outcomes. This design is offered as a testable plan, and its evaluation would provide the empirical validation that the framework still requires.

## 6. Conclusions and Future Directions

This framework deliberately prioritizes institutional and economic mechanisms over technological ones. Portable detection technologies, digital traceability tools, and smartphone-based diagnostics hold genuine promise [52,53], but without complementary investment in incentive alignment, capacity building, and governance, they risk low uptake or even exacerbating inequality by disproportionately benefiting better-resourced actors. A related tension concerns collective action: while aggregation through cooperatives is often promoted to strengthen smallholder bargaining power, mixing grain from multiple producers can dilute quality signals and compromise traceability. The framework addresses this by placing monitoring at the aggregation handoff, before mixing occurs, thereby preserving both the economic advantages of collective action and the integrity of quality information required for incentives to function. Underlying both points is a design principle that should guide any intervention in these systems: will it continue to function once the introducing project ends? Market premiums, institutional procurement standards, professionalized local service providers, and community-based governance are emphasized throughout this framework specifically because they embed monitoring in economic logic rather than donor-dependent timelines.

Two limitations of the incentive logic should be stated directly. First, intermediaries who optimize for turnover may mask contamination by blending highly contaminated grain with sound lots. This behavior spreads contamination and can defeat a handoff test performed only once. The framework mitigates it by testing at the aggregation handoff before mixing occurs, by re-testing on the buyer side at each subsequent handoff, and by making “Verified Seller” status a reputational asset that may be forfeited if downstream testing exposes masking. Where no downstream testing or reputational stake exists, however, masking is likely to persist. Second, the incentive model does not rely on consumer willingness to pay a premium in low-income retail markets, where price and availability dominate. Its leverage is instead buyer- and institution-side, operating at the point of transaction and through procurement standards rather than through voluntary condemnation of grain by the actor who holds it.

The movement of grain through aggregation and cross-border mixing also has a network character, in that contamination and risk may diffuse across connected nodes, shaped by proximity and connectivity. A full network model is beyond the scope of this conceptual framework. Network-based analysis of product movement is a valuable direction for future quantitative work [54].

Several research priorities would strengthen the evidence base for implementation. First, field validation of Tier 1–2 tools is needed, including head-to-head comparison of lateral flow assays (LFA), smartphone-based readers, and BGYF methods against reference standards across diverse crops and agroecological conditions. The aim is to assess reliability and accuracy under real-world DAS contexts. Second, cost-effectiveness modeling of incentive structures, including the minimum price premium required to shift stakeholder behavior or the return on investment for contamination mitigation products (e.g., hermetic storage), would inform program design. Discrete-choice experiments and willingness-to-pay or willingness-to-test studies would provide direct evidence on the stakeholder responses assumed in the framework. Third, longitudinal studies tracking monitoring adoption, sustainability, and health outcomes in communities where the framework is piloted would generate critical evidence for scaling. Finally, systematic assessment of applicability to contaminants beyond aflatoxins, including other mycotoxins, heavy metals, pesticide residues, and microbial contamination, and to additional crops beyond maize would test the framework’s intended generalizability. These hazards differ from mycotoxins in origin, kinetics, detection method, and the location of their control points, and they are therefore likely to require hazard-specific adaptation rather than direct transfer of the aflatoxin-derived control points.

Decentralized agricultural systems feed hundreds of millions of people in some of the world’s most vulnerable communities, yet they remain outside the reach of monitoring frameworks designed for centralized food systems. Closing this gap requires not the extension of centralized models but a framework adapted to the structural realities of DAS: dispersed infrastructure, household-level operational burdens, informal economic dynamics, and fragmented governance. The four principles presented here—tiered monitoring, handoff-point placement, self-sustaining incentives, and durable capacity building and governance—offer a structured approach grounded in the insight that monitoring need not function as a regulatory burden imposed on already-constrained actors. When embedded at strategic nodes and aligned with appropriate incentives, monitoring becomes a source of value: higher and more stable prices for producers, reduced risk for buyers, verifiable outcomes for institutional programs, and safer food for consumers.

Future implementation in contexts like Guatemala could involve piloting the model within school feeding procurement, aligning institutions and cooperatives around quality standards, and testing hybrid verification systems. Incentive-based purchasing and capacity building may support adoption, while gradual institutionalization within procurement frameworks could enable potential scale-up beyond donor-supported initiatives.

## Figures and Tables

**Table 1:** Decentralized Agricultural Systems (DAS) supply chain typology; 10 scenarios characterized by key process steps, stakeholders, scale, timing, and quality control (QC) practices. Storage durations, scales, and volumes are illustrative expert estimates for the Guatemalan context rather than measured values; entries supported by citation are referenced as such.

Scenario/End Point	Key Steps in the Chain	Stakeholders Involved	Typical Storage & Transport Time (Days)	Typical Scale/Volume	Common Handling/Quality Control Practices
**1. Direct Household Consumption**	- Smallholder farmers grow maize on family plots. - Maize is harvested, dried, stored (often on-farm). - The family uses stored maize for home consumption (tortillas, tamales, etc.).	- Smallholder farmers - Household members	On-farm storage can range from weeks to months; transport minimal or none (home use).	Typically small (e.g., sacks of 50–100 kg).	- Traditional sun-drying on patios or corncobs stored in the rafters. - Minimal formal checks (visual inspection).
**2. Direct Sales to Local/Small Markets**	- Households/farmers grow maize. - Harvest, dry, store on-farm (short-term). - Farmers transport their own maize to local/community market.	- Smallholder farmers - Local market vendors - Local consumers	1–5 days total from harvest to sale if quickly sold; short on-farm storage (~1–2 weeks).	Small to medium (~100 kg to a few hundred kg).	- Often visual checks only. - Sun drying or basic bagging for transport.
**3. Informal Group Aggregation for Local Markets**	- Neighboring farmers pool their maize post-harvest. - One or more representatives transport the aggregated maize to local market. - Sales are typically face-to-face.	- Smallholder farmers - Informal farmer groups or co-ops - Market buyers and local consumers	Pooled storage can last 1–2 weeks before transport; transport itself typically 1 day or less.	Small to medium (possibly up to 1 ton total if multiple farmers pool).	- Basic checks by group members (e.g., removing visibly moldy cobs). - Usually bagged in polypropylene sacks.
**4. ‘Middleman’ Aggregation for Local Markets**	- Farmers harvest and dry maize. - Middlemen travel to farms to purchase maize (possibly mixing sources, including imports from Mexico). - Middlemen sell to local markets.	- Smallholder farmers - Middlemen - Local market vendors - Local consumers	1–3 days from on-farm to local market once purchased. Middlemen often do quick turnover.	Medium (a few tons aggregated from several farmers).	- Some informal sorting to remove obviously moldy grain. - No formal moisture testing typically.
**5. ‘Middleman’ Aggregation for Larger Regional Markets**	- Farmers sell directly to middlemen. - Maize is aggregated from multiple farms/regions (may include imports). - Sold at larger municipal/regional markets or via secondary traders.	- Smallholder farmers - Middlemen - Transporters - Warehouse owners - Larger market vendors	Storage can be up to 1–2 weeks in middlemen’s warehouses before further transport.	Medium to large (several tons or more).	- Might check moisture by touch or taste; rarely any lab testing. - Bagged storage in small depots.
**6. Maize Sold to Local Restaurants/Tortillerías**	- Farmers or middlemen sell maize to local eateries (small restaurants, tortillerías). - Establishments process maize on-site (nixtamalization, grinding).	- Smallholder farmers - Middlemen - Restaurant/tortillería owners - Local consumers	Often 1–2 days between purchase and use; minimal storage due to frequent turnover.	Small (daily or weekly needs).	- Visual inspection on delivery; occasional smell check. - Nixtamalization can reduce some mycotoxins.
**7. Supply to Local Milling/Processing Plants**	- Farmers or middlemen deliver maize to small or medium-scale mills. - Maize may be milled into flour, packaged, and distributed locally.	- Smallholder farmers - Middlemen or co-ops - Mill operators - Local retailers (corner stores) - Consumers	Storage at mills can be up to a few weeks, depending on demand. Transport time usually 1–2 days.	Medium volumes (hundreds of kg to a few tons).	- Basic moisture checks and sometimes weigh stations at mills. - Bagging and short-term silo storage.
**8. Supply to Larger Food Processing Facilities**	- Farmers or aggregators sell maize in bulk to larger commercial mills or processing plants. - Processed products (flour, snack foods, etc.) are distributed nationally or regionally.	- Smallholder farmers - Aggregators (co-ops or private) - Large processing facility managers - Grocery stores	Bulk storage can be weeks to months in large silos. Transport might involve 2–3 days between regions.	Large (multiple tons).	- Often have formal checks (moisture content, sometimes basic mold testing). - Controlled storage (temperature, humidity).
**9. Institutional/Gov’t Procurement & Distribution**	- Farmers or aggregators sell maize to government entities (for school feeding programs, social assistance, etc.). - The maize may be stored in gov’t warehouses before distribution.	- Smallholder farmers - Aggregators - Government agencies - Schools/NGO programs	Could be long-term storage (months) in government warehouses if distribution is delayed.	Large (depending on program size).	- May have procurement standards (some moisture checks). - Varies by program; not always enforced strictly.
**10. Cross-Border Trade (Informal or Formal)**	- Maize enters Guatemala from neighboring countries (e.g., Mexico). - Imported maize is sold to local middlemen or distributors. - Ends up in local or larger markets, restaurants.	- Foreign maize exporters - Border agents (formal or informal) - Middlemen/Aggregators - Market/restaurant buyers	Storage times vary widely (days to weeks), especially if crossing the border informally.	Medium to large (can be small trucks to large freight).	- Spot checks at formal crossings; minimal checks at informal routes.

**Table 2:** Stakeholder matrix summarizing roles, motivations, constraints, and the proposed value propositions for monitoring adoption.

Stakeholder	Roles/Activities	Motivations/Compensation	Constraints/Barriers to Improved Quality
**Smallholder Farmers**	- Cultivate maize on family plots. - Harvest, dry, and store maize. - May sell surplus to markets or middlemen. - Often also use maize for home consumption.	- Income from surplus sales. - Food security (subsistence). - Minimize losses to mold/pests.	- Limited access to improved storage (e.g., hermetic bags). - Financial constraints (lack of credit/insurance). - Low awareness of aflatoxin mitigation practices.
**Household Members (Home Consumers)**	- Primarily store and prepare maize for family meals. - May sell small surplus or buy additional maize if needed.	- Feeding family (quality & safety). - Cost savings (vs. buying retail).	- Minimal time/incentive to test own maize. - Lack of knowledge about proper moisture levels. - Unpredictable weather or pests affecting home-stored maize.
**Informal Farmer Groups/Co-ops**	- Pool/aggregate maize from members. - Share resources for transport, negotiation, or storage. - May coordinate sales to markets or middlemen.	- Better bargaining power (larger volumes can get better prices). - Reduced transport costs. - Possible group support (shared funds).	- Group cohesion can be weak if trust is low. - Lack of formal quality control or testing. - Storage facility costs may be high, requiring external funding.
**Local Market Vendors**	- Purchase maize from farmers or middlemen. - Sell maize in small quantities to consumers. - Minimal on-site storage.	- Profit from retail margin. - Stable supply and quality to keep local customers.	- Low capital to invest in better storage/handling. - Little incentive for formal testing if customers are not demanding it.
**Middlemen/Aggregators**	- Travel to farms to buy maize in bulk. - Transport maize to local/regional markets or to processors. - Often mix maize from various sources (including imports).	- Profit from price differentials (buy low, sell higher). - Efficiency in logistics.	- No direct premium for higher-quality grain, so limited incentive. - Often focused on quick turnover vs. quality. - Informal networks complicate traceability.
**Local Restaurants/*Tortillerías***	- Purchase maize (often small, frequent batches). - Process/cook maize on-site. - Sell prepared foods directly to consumers.	- Profit from selling food. - Consistent supply & quality for repeat customers.	- Daily or weekly purchases reduce large-scale storage but also hamper systematic quality checks. - Little awareness of potential liability for poor-quality maize.
**Local/Community Mills**	- Provide milling services for a fee. - May buy maize to sell flour locally. - Typically small or medium scale.	- Service fees (paid by farmers/households). - Profit from re-selling milled flour.	- Lack of testing equipment or training to detect aflatoxins. - Low margins make investing in improved storage/quality checks difficult.
**Larger Commercial Mills/Processing Facilities**	- Buy maize in bulk from farmers or aggregators. - Process into flour or other products. - Sell regionally or nationally.	- Profit from value-added products. - Brand reputation can matter. - Long-term contracts require steady supply.	- High capital costs for advanced storage and testing might not be offset if consumer regulations are not enforced. - Dependence on aggregator reliability/quality.
**Government Agencies & Programs**	- Purchase maize for social programs (schools, food aid). - May operate warehouses for distribution. - Can set or enforce quality standards (though enforcement may vary).	- Public welfare & food security. - Cost-effectiveness in procurement. - Political/social objectives (support local farmers).	- Bureaucratic delays can cause long storage periods. - Limited enforcement of standards if resources are lacking. - Budget constraints can limit advanced testing.
**NGOs/Development Organizations**	- Provide training on best practices (e.g., improved storage, post-harvest handling). - May organize co-ops or offer microfinance.	- Mission-driven to improve livelihoods & reduce food insecurity. - Donor obligations for program outcomes.	- Dependence on donor funding which can be short-term. - Challenges in scaling successful pilot interventions regionally. - Coordination with government or private sector can be complex.
**Transporters/Truck Drivers**	- Move maize from farms to markets, or from middlemen to mills/warehouses. - May operate informally or under contract.	- Payment per trip/volume transported. - Maintaining repeat business by reliability.	- No direct incentive to ensure quality in transit (focus is speed). - Poor road conditions or infrastructure can prolong transit, increasing risk of spoilage.
**Border Agents (Formal/Informal)**	- Inspect maize crossing the border (formal). - May facilitate or ignore unregulated imports (informal).	- Salary (official). - Bribes in corrupt scenarios.	- Weak regulation or oversight can enable illegal imports. - Minimal training in detecting mold or contamination. - Corruption undermines formal controls.
**Foreign Maize Exporters**	- Grow or aggregate maize in neighboring countries. - Sell it across borders (formal or informal).	- Profit from exporting to demand-driven markets. - Volume-based income (more shipments, more revenue).	- Different regulations in source countries. - Limited responsibility once product crosses the border. - Fluctuating border policies can disrupt business.
**Warehouse Owners/Operators**	- Provide storage space for aggregated maize (private or co-op owned). - Charge fees for storage services.	- Storage fees from users. - Profit depends on high utilization and timely turnover.	- Upfront investment in proper ventilation or climate control can be expensive. - No direct impetus to test if clients do not demand it. - Risk of spoilage if maintenance is poor.

## Data Availability

No new data were created or analyzed in this study.

## Supplementary Materials

The following supporting information can be downloaded at https://www.mdpi.com/article/10.3390/toxins18080335/s1: Table S1: Comparative characterization of CAS and DAS across physical, economic, social, and regulatory dimension. Text S1: A robust framework for monitoring in DAS requires a precise characterization of what these systems are and how they differ structurally from the centralized systems for which existing monitoring frameworks were designed. This section draws on the four-dimensional characterization developed for decentralized water systems–physical, economic, social, and regulatory–and adapts it to the agricultural domain.
