# Supplementary material for: A Framework for Monitoring Contaminants in Decentralized Agricultural Systems: Leveraging Stakeholder Incentives in Low-Resource Settings
Source: Toxins (Basel). 2026 Aug 1;18(8):335. doi: 10.3390/toxins18080335 (PMC13517755; doi:10.3390/toxins18080335)
Supplement: Supplementary file 1 [file toxins-18-00335-s001.zip › toxins-4381282-supplementary.pdf]

Supplementary Materials: A Framework for Monitoring Contaminants in Decentralized Agricultural Systems: Leveraging Stakeholder Incentives in Low-Resource Settings

Lee E. Voth-Gaeddert, Hannah Glesener and Gabriela Montenegro-Bethancourt

Supplemental Table S1. presents a comparative summary of the key structural differences between centralized and decentralized agricultural systems across the four dimensions discussed above. These differences are not presented as deficiencies of DAS to be corrected but as structural features that any monitoring framework must accommodate.

Table S1. Comparative characterization of CAS and DAS across physical, economic, social, and regulatory dimensions

**Dimension**	**Centralized Agricultural Systems (CAS)**	**Decentralized Agricultural Systems (DAS)**
**Physical infrastructure**	Large-scale production, industrial processing, controlled-environment storage, formalized transport	Small plots, traditional drying/storage (patios, tapancos, sacks), informal transport (foot, motorbike, small truck)

**Operational burden**	Managed by specialized staff with standard operating procedures	Borne primarily by individual households across all post-harvest stages
**Economic structure**	Centralized finance, formal contracts, access to credit/insurance, brand reputation incentivizes quality	Household-borne costs, informal transactions, middlemen prioritize volume over quality, no quality premium
**Social dynamics**	Formal relationships between corporate entities and supply chain partners	Dependent on informal networks for knowledge, resources, and collective action; co-ops lack formal QC
**Regulatory oversight**	Defined federal/state regulations, formal standards, certification, and enforcement	Fragmented, vaguely defined, often unenforced; minimal explicit frameworks for informal DAS
**Traceability**	Formal tracking systems, high transparency	Limited; mixing by intermediaries severely compromises tracking
**Quality incentives**	Strong market/regulatory incentives; testing is routine	Weak; no premium for quality; testing is rare; quality based on trust

Supplemental Text S1:

A robust framework for monitoring in DAS requires a precise characterization of what these systems are and how they differ structurally from the centralized systems for which existing monitoring frameworks were designed. This section draws on the four-dimensional characterization developed for decentralized water systems — physical, economic, social, and regulatory — and adapts it to the agricultural domain.

S1.1 Defining DAS Along the Centralized–Decentralized Spectrum

Agricultural production and distribution exist along a continuum. At one end, centralized agricultural systems (CAS) are characterized by large-scale monoculture production, industrial processing facilities, extensive and formalized transport networks, controlled-environment storage, and comprehensive regulatory oversight. At the other end, DAS involve geographically distributed networks of small-scale production, informal processing and storage, and localized consumption, often with minimal formal regulation.

It is important to note that DAS are not simply "small-scale CAS" nor reducible to subsistence farming. Subsistence farming — in which nearly all output is consumed by the producing household — represents one configuration within DAS, but the category also includes semi-commercial producers who sell surplus, cooperatives that aggregate output for collective marketing, and informal chains in which intermediaries connect producers to local and regional markets. These varied configurations share a common structural feature: the absence of centralized infrastructure, management, and oversight, which in turn places the burden of quality assurance on the actors least equipped to bear it. Understanding DAS as a spectrum of

configurations, rather than a monolithic category, is essential for designing monitoring approaches that are calibrated to the specific structures and scales of different chain types.

S1.2 Physical Components and Operational Realities

Unlike CAS, which rely on purpose-built infrastructure managed by specialized entities, DAS are constituted by dispersed, often household-owned physical components. Agricultural plots are typically small (often less than two hectares), and drying, storage, and initial processing take place using traditional methods: sun-drying on patios, storage of cobs in kitchen lofts (*tapancos*) or in polypropylene sacks, and transport by foot, motorbike, or small truck.

This dispersed infrastructure imposes a high operational burden on individual farmers and households, who are directly responsible for managing the critical post-harvest stages — harvesting, drying, storage, and transport — that most strongly determine contaminant risk. Traditional sun-drying, for instance, is highly susceptible to variation in moisture management; inadequate drying creates favorable conditions for fungal colonization and mycotoxin production. The challenge is compounded by limited access to improved technologies: hermetic storage bags, which have been shown to reduce aflatoxin contamination by approximately 53% over three months in field trials in Kenya, remain beyond the reach or awareness of many smallholders. A framework for DAS must therefore address how to introduce accessible, maintainable physical improvements and operational practices that reduce individual burden without requiring centralized infrastructure.

S1.3 Economic Structure

The economic dynamics of DAS differ fundamentally from those of centralized systems. Individual households bear the direct costs of operation, maintenance, and any quality-related investments, with limited access to formal credit, insurance, or grant mechanisms. The primary economic motivations for smallholder producers are household food security and income from surplus sales — objectives that may conflict with quality improvement when contaminated grain can still be consumed or sold at a lower price.

A distinctive economic actor in many DAS is the intermediary or middleman — known as *coyotes* in the Guatemalan context — who purchases maize in bulk from multiple producers, frequently mixing sources, and resells to markets or processors. Middlemen are motivated by price differentials and rapid turnover, and they generally lack incentives to invest in quality testing or to pay premiums for higher-quality grain. Their purchasing practices, particularly the mixing of maize from diverse sources, actively undermine traceability and dilute any quality signals that individual producers might otherwise convey to downstream buyers.

At the same time, individual households in DAS are generally less equipped than formal enterprises to access the financial mechanisms that could support quality improvement. The result is a structural disincentive: the actors who would benefit most from quality improvement (consumers, institutional buyers) are separated from the actors who control quality outcomes (producers, intermediaries) by economic arrangements that do not reward quality and may even penalize it, as higher-quality grain takes more time, effort, and investment to produce.

S1.4 Social Dynamics

The resilience and functionality of DAS depend heavily on informal social networks. Information about farming practices, post-harvest handling, equipment performance, and market conditions is exchanged through kinship ties, neighborhood relationships, and informal farmer groups. These networks facilitate resource pooling (e.g., collective transport to market), mutual aid, and the diffusion of innovations, and they constitute a form of social capital that is both a strength and a constraint.

Informal farmer groups or cooperatives may aggregate maize post-harvest and coordinate transport and negotiation, offering efficiencies in transaction costs and potentially enhancing bargaining power. However, these groups typically lack formal quality control mechanisms, standardized testing capabilities, or institutional structures that would enable systematic quality assurance. The social cohesion that enables collective action can also impede it: where trust is low, group norms around quality may be difficult to establish, and the mixing of grain from members of varying quality erodes individual accountability.

S1.5 Regulatory Landscape and Governance

The regulatory environment for DAS is characterized by fragmentation and neglect. Regulations governing agricultural quality and safety are often vaguely defined, inconsistently enforced, and designed primarily for centralized, formal-sector supply chains. Even in the United States, the USDA administers aflatoxin certification programs for specific export commodities (almonds, peanuts, pistachios) and offers Good Agricultural Practices (GAP) audit services, but these programs target formal market access and do not address the realities of

informal, decentralized systems. In low-income countries, the regulatory gap is wider: standards may exist on paper but lack the institutional capacity, funding, and local presence needed for enforcement.

This regulatory vacuum is compounded by what has been described as an "urban bias" in agricultural policy, which directs resources and attention toward centralized, commercially oriented systems and overlooks the needs of rural, decentralized producers. The consequence is that DAS operate largely outside formal governance structures, leaving quality assurance to informal norms and individual practice. Any framework for monitoring integration must therefore work within this governance reality — proposing mechanisms that can function without strong regulatory enforcement while also identifying opportunities for incremental policy innovation.
